# Prevalence of hypertension among federal ministry civil servants in Addis Ababa, Ethiopia: a call for a workplace-screening program

**DOI:** 10.1186/s12872-015-0062-9

**Published:** 2015-07-22

**Authors:** Kassawmar Angaw, Abel Fekadu Dadi, Kefyalew Addis Alene

**Affiliations:** Addis Ababa, Ethiopia; Institute of Public Health, College of Medicine and Health Sciences, University of Gondar, Gondar, Ethiopia

**Keywords:** Hypertension, Ministries civil servants, Ethiopia

## Abstract

**Background:**

The prevalence of hypertension (HTN) is increasing rapidly in Ethiopia, but data are limited on hypertension prevalence in specific workplaces. Therefore, the aim of this study was to assess the prevalence and associated factors of hypertension among federal ministry civil servants.

**Methods:**

Institutional based cross sectional study was conducted from February to April 2014. Simple random sampling technique was used to select 655study participants. A standardized questionnaire adapted from The World Health Organization’s (WHO) STEP tool was used to collect the data. In this study, HTN was defined as mean systolic blood pressure (SBP) and diastolic blood pressure (DBP) of 140/90 mmHg and above, and patients on regular drug therapy for H. Data were entered into EPI-Info 3.5.2 and analyzed by SPSS version 20. Binary logistic regression model was used to identify associated factors. Odds ratio with 95 % CI was computed to assess the strength of the association and significant level.

**Result:**

The prevalence of hypertension was found to be 27.3 % (95 % CI 23.3 – 31 %). Civil servants of age 48 years and above [AOR = 5.88, 95 % CI: 2.36-14.67], age 38-47 years [AOR = 2.80, 95 % CI: 1.18-6.60] and age 28-37 years [AOR = 2.35, 95 % CI: 1.00-5.56]) were more likely to be hypertensive. Similarly, ever cigarette smoking [AOR =2.34(1.31-4.17), family history of hypertension [AOR = 3.26, 95 % CI 1.96-5.40], self-reported Diabetes Mellitus (DM) [AOR = 13.56, 95 % CI: 6.91-26.6], and body mass index (BMI > 25 kg/m^2^) [AOR = 7.36, 95 % CI: 2.36-14.67] were found to be significantly associated with hypertension.

**Conclusion:**

The prevalence of hypertension among federal ministry civil servants was found to be high; which is an indication for institution based hypertension-screening programs especially focusing on those aged 28 years and above, obese, DM patients and cigarette smokers.

## Background

Hypertension is a chronic medical condition in which the blood pressure in the arteries is elevated. It contributes to the burden of heart disease, stroke, kidney failure, and premature death and disability. Hypertension rarely causes symptoms in the early stages and many people are undiagnosed [1].

The proportion of the global burden of disease attributable to hypertension has significantly increased from 4.5 % in the year 2000, to 7 % in 2010 [2]. Early diagnosis of hypertension is important in order to avoid potentially life threatening complications. Hypertension is also high in people with risk factors like tobacco use, physical inactivity, unhealthy diet, obesity, diabetes, high cholesterol, low socioeconomic status and family history of [3].

The WHO’s 2012 report highlighted the growing burden of non-communicable diseases worldwide. Hypertension causes around half of all stroke and heart disease related deaths. About 7.1 million or 6 % of deaths worldwide was attributed to hypertension. High blood pressure (BP) is responsible for 62 % of cerebrovascular disease and 49 % of ischemic heart disease [2, 3].

Hypertension was once thought to be rare in Africa, but it is now recognized as one of the most common causes of cerebrovascular diseases accounting for about 40 % of cerebrovascular diseases on the African continent [4]. There is an urgent need to develop strategies to prevent, diagnose, and treat hypertension more effectively in Africa [4]. Today, there are approximately 80 million adults with hypertension in Africa and projections based on current epidemiological data suggest that this figure will rise to 150 million by 2025 [5].

A systematic review revealed that hypertension is one of the most important public health issues in sub-Saharan Africa (SSA), particularly in urban areas, with evidence of considerable under-diagnosis, inadequate treatment and control. Despite their growing prevalence in SSA hypertension and other cardiovascular disease were not given due attention. An increasing burden of hypertension in this region is likely to result in increasingly poor outcomes, as very few people receive adequate treatment, and achieve their goal BP [6].

The prevalence of hypertension has been steadily increasing in Ethiopia from 3.6 % in 1983, to 11.8 in 2002 and 29.6 in 2006 [7]. Even though work related prevalence study is limited in the country, prevalence of hypertension is around 30 % in some urban areas in Ethiopia [8, 9]. Currently there is also no occupational health policy in Ethiopia. This makes hypertension the most important problem that needs urgent action. The prevention and control of high blood pressure has not received adequate attention in many developing countries like Ethiopia.

Hypertension has been widely studied in various regions of the world. Even though hypertension studies have been conducted in Ethiopia, its relationship with specific occupations has not been established. WHO recommends workplace wellness program with a focus on health promotion through the reduction of individual risk-related behaviors like; tobacco use, unhealthy diet, excessive alcohol use, and physical inactivity [10].

Therefore, additional information is needed to build evidence for the development of health improvement activities in Ethiopian civil servants. The main aim of this study was to assess the prevalence of hypertension and its associated factors among ministry civil servants in Addis Ababa. We hope this can provide information regarding prevalence of hypertension and associated factors for those concerned with developing workplace health programs in Ethiopia.

## Methods

### Study design and setting

Institutional based cross sectional study was conducted from February to April 2014 among Addis Ababa ministry civil servants. Addis Ababa is the capital city of Ethiopia, and the headquarters of the African Union and several international organizations. It has ten sub cities and 116 districts with a total populations of 3.5 million [11]. The Federal Democratic Republic of Ethiopia is composed of 21 ministries. All the ministries are centered in Addis Ababa and there are around 15,808 civil servants under all ministries combined (Ministry of civil Services roport on number of civil servantes in all ministries in Ethiopia. In: Human resourece Department, Addis Ababa 2012, unpublished report). The city has 18 hospitals, 24 health centers, 161 health stations, 340 clinics and 47 health posts run by government, private, and non-governmental organizations (NGOs).

### Study population and sampling procedure

The study population was all civil servants who are working at ministries. Those ministry civil servants who were pregnant during the data collection period were excluded. The necessary sample size (n) was computed by single population proportion formula [n = [(Z_a/2_)^2^*P (1-P)]/d^2^] by assuming 95 % confidence level of Z a/_2_ = 1.96, margin of error 5 %, design effect 2, proportion (p) of HTN 28.3 % according to the previous similar study from Gondar city [9], and non-response rate 5 %. The calculated sample was 655.

Multi stage sampling technique was employed to select the study participants. Four ministries; Ministry of Education, Ministry of Culture and Tourism, Ministry of Urban Construction, and Ministry of Civil Service were randomly selected from the twenty one ministries of the Ethiopian Federal government. The civil servant lists were obtained from the human resource department of the respective ministries. Participants were randomly and proportionally selected from the list of each ministry. When a selected individual was unable or unwilling to participate, a replacement was randomly selected from the list and interviewed.

### Data collection and diagnosis of hypertension

A standardized questionnaire adapted from the WHOSTEP tool was used to collect the data [12]. Socio demographic (sex, age, marital status, religion educational status), behavioral (smoking, alcohol consumption, chat chewing, physical activity, dietary habit), and other variables like DM status, family history of hypertension were collected. The questionnaire was self-administered. Weight and height measurements were taken to determine body mass index. Weight was measured, in kilograms using a bathroom weighing scale with the subjects standing, arms hanging naturally at the sides, without footwear. Height was measured, in meters, using a stadiometer, to the crown of the head, the subject standing without any footwear or headgear and looking straight ahead.

Body-mass index (BMI) was calculated as weight in kilograms over height in meters squared [weight (kg)/ (height (M)) ^2^]. The study participants were considered underweight when their BMI was below 18.5 kg/cm^2^, normal when their BMI was between 18.5 kg/M^2^ and 24.9 kg/M^2^, overweight when their BMI was between 25 kg/M^2^ and 29.9 kg/M^2^ and obese when their BMI was greater than or equal to 30 kg/M^2^.

Blood pressure (BP) was measured by four BSc degree Nurses in a sitting position using a standard mercury sphygmomanometer BP cuff with the appropriate cuff size that covers two-thirds of the upper arm and stethoscope. The cuff was inflated at rate 2-3 mmgh/second and the first Korotkoff sound indicated systolic BP and the diastolic BP was recorded when the sound disappeared. Before measurement, participants were asked to rest for at least five minutes. They were asked to confirm that they had not smoked or consumed caffeine-containing products for at least 30 min before measurements. Two consecutive measurements were taken in an interval of at least 5 min. Mean systolic and diastolic blood pressures were determined by averaging the first and second measurements (34). In this survey, hypertension was defined as mean systolic blood pressure (SBP) and diastolic blood pressure (DBP) of 140/90 mmHg or greater, and Hypertensive patients on regular drug therapy for hypertension. This is based on the definition of the European Society of Hypertension (ESH), European Society of Cardiology (ESC),WHO and International Society of Hypertension guidelines (ISH) [12–14].

### Data analysis

Data were entered to Epidemiological Information (EPI-INFO) software version 3.5.1 and analyzed by Statistical Package for Social Sciences (SPSS) software version 20. Descriptive statistics such as frequency and cross tabulation were calculated for selected variables. A backward binary logistic regression model was used to identify factors associated with hypertension. Both Crude Odds Ratio (COR) and Adjusted Odds Ratio (AOR) with 95 % confidence interval (CI) were used to show an association between hypertension and selected variables. Variables having p-value ≤ 0.05 in the final model were assumed significant determinants. Model fitness test was evaluated by the Hosmer and lemeshow goodness of fit test.

### Ethical consideration

Ethical clearance was obtained from the Institutional Review Board of University of Gondar. Official letters were obtained from administrative body of each ministry. The purpose of study was explained to the study participants and informed written consent was obtained. Confidentiality was maintained at all levels of the study by avoiding use of names and other identifiers. Those study participants found hypertensive were advised and linked to the nearby health institution for further diagnosis and treatment.

## Results

Six hundred twenty nine ministry civil servants (with a response rate of 96 %) participated in the study and of which 279 (44.4 %) were male. Their mean (±$SD$) age was 38.96 (±10.41) years. Majority of the participants were Orthodox Christianity followers (458, 72.8 %). Three hundred and seventy four respondent were married (374, 59.5 %) and 408 (64.9 %) were educated to the level of diploma and above. (Table 1).

**Table 1:** Socio-demographic characteristics of respondents among ministries civil servants in Addis Ababa, Ethiopia, 2014 (n = 629)

Variable	Category	Frequency	%
Sex	Male	279	44.4
	Female	350	55.6
Age	18-27	138	21.9
	28-37	214	34
	38-47	167	26.6
	≥48	110	17.5
Religion	Orthodox	458	72.8
	Muslim	59	9.4
	Protestant	97	15.4
	Others	15	2.4
Marital status	Single	213	32.9
	Married	374	59.5
	Divorced	21	3.3
	Widowed	21	3.3
Educational level	Write and read only	29	4.6
	primary education (1-8)	70	11.1
	secondary education (9-12)	122	19.4
	Diploma and above	408	64.9
monthly income (ETB)	<1000	119	18.9
	1000-2250	250	39.7
	2251-3300	102	16.2
	= > 3301	158	25.1

About half, 316 (50.2 %) and one-fourth, 117 (18.6 %) of the participant reported their past or present alcohol use and use of cigarettes, respectively. From these 248 (78.5 %) and 30 (25.6 %) were current alcohol users and cigarette smokers, respectively. One hundred six (16.9 %) of the respondents were reported chewing chat in the past. Majority 582 (93.6 %) of the respondents consumed fruit 1-3 days per week and 501 (80.2 %) of the participants reported their vegetable consumption 1-3 times per week. (Table 2)

**Table 2:** Behavioural and dietary habit of respondents among ministries civil servants in Addis Ababa, Ethiopia, 2014

Variable	Category	Frequency	Percent (%)
Cigarette smoking habit (n = 629)	Never	512	81,4
	Current	30	4.8
	Previous	87	13.8
Ever consumed an alcoholic drink (n = 629)	Yes	316	50.2
	No	313	49.8
Alcohol consumption frequency in a week (n = 316)	Daily	10	3.2
	5-6	19	6
	3-4	39	12.3
	1-2	248	78.5
Chat chewing practice (n = 629)	never	523	83.1
	current	31	5
	previous	75	11.9
Fruit eating habit per week (n = 622)	1-3 days	582	93.6
	4-7 days	40	6.4
Vegetable consumption frequency per week (n = 625)	1-3 days	501	80.2
	4-7 days	124	19.6
Types of oil or fat consumed (n = 629)	Vegetables	250	39.7
	Butter	69	11
	Sesame/nug oil	267	42.4
	Others	43	6.8

About one third (30.8 %) of the respondents were involved in vigorous-intensity physical activity. The majority (84.6 %) of the study participants walked at least 10 min per day and 88 (14 %) were ever told they had DM. One-fourth (27.3 %) of the study participants were overweight or obese (Table 3).

**Table 3:** Factors associated with hypertension among ministries civil servants in Addis Ababa, Ethiopia, 2014

Variable	Hypertension		COR (95 % CI)	AOR (95 % CI)
	Yes	No		
Age of the participant				
18-27 Years	10 (7.2 %)	128 (92.8 %)	1	1
28-37 Years	49 (22.9 %)	165 (77.1 %)	3.8 (1.85-7.80)	2.35 (1.00-5.50)
38-47 years	53 (31.7 %)	114 (68.3 %)	5.95 (2.89-12.24)	2.80 (1.18-6.60)
≥48 Years	60 (54.5 %)	50 (45.5 %)	15.36 (7.29-32.35)	5.88 (2.36-14.67)
Ever cigarette smoking				
Smoker	62 (53 %)	55 (47 %)	4.12 (2.70-6.27)	2.34 (1.31-4.17)
Non smoker	110 (21.5 %	402 (78.5 %)	1	1
Ever alcohol drinking				
Yes	104 (32.9 %)	202 (67.1 %)	1.77 (1.24-2.53)	
No	68 (21.7 %)	245 (78.3 %)	1	
Ever chat chewing status				
Yes	38 (35.8 %)	68 (64.2 %)	1.62 (1.04-2.53)	
No	134 (25.6 %)	389 (74.4 %)	1	
Family history of HPN				
Yes+	78 (46.7 %)	89 (53.3 %)	3.49 (2.38-5.12)	3.26 (1.96-5.40)
No	90 (20 %)	359 (80 %)	1	1
Ever told had DM				
Yes	66 (75 %)	22 (25 %)	12.31 (7.27-20.86)	13.56 (6.91-26.60)
No	106 (19.6 %)	435 (80.4 %)	1	1
BMI				
Normal Weight	60 (15 %)	341 (85 %)	1	1
Under Weight	7 (12.5 %)	49 (87.5 %)	0.812 (0.35-1.88)	0.54 (0.19-1.52)
Overweight/obese	105 (61 %)	67 (39 %)	8.90 (5.90-13.44)	7.36 (4.43-12.23)

### Prevalence of hypertension and associated risk factors

The mean SBP and DBP were 125.96 ± 13.93 mmHg and 81.44 ± 10.46 mmHg, respectively. The overall prevalence of hypertension was 27.3 % (*95 % CI:* 23.8-31.0); of which 28.3 % were females and 26.2 % were males (p-value >0.5). Accordingly, based on ESH classification, five (0.8 %) civil servants had isolated systolic hypertension (> = 140/<90 mmHg), 151(24.01 %) had grade I hypertension, 13(2.1 %) had grade II hypertension and three (0.5 %) had grade three hypertension. The blood pressure distribution for both systolic and diastolic is normal (Kolmogorov-Smirnov test, p-value <0.0001).

In the multivariate logistic regression analysis, older age, cigarette use, family history of hypertension, self-reported DM, and BMI > 25 kg/m^2^ were significantly associated with Hypertension. Accordingly, the prevalence of hypertension was increased with age. Participants who were age 48 years and above [AOR = 5.88, 95 % CI (2.36-14.67)], 38-47 years [AOR = 2.80, 95 % CI: 1.18-6.60] and 28-37 years [AOR = 2.35, 95 % CI: 1.00-5.56] were more likely to be hypertensive as compared to those who were in age category of 18-27 years.

Past or present cigarette smokers are 2.34 times more likely to have hypertension as compared to non-smokers (AOR = 2.34, 95 % CI: 1.31-4.17). Similarly, study participants who had family history of hypertension [AOR = 3.26, 95 % CI (1.96-5.40)] and self-reported DM [AOR = 13.56, 95 % CI (6.91-26.60)] were 3.26 and 13.56 times more likely to be hypertensive respectively. In addition to these, study participants who were overweight/obese were 7.36 times more likely to be hypertensive as compared to those who had normal BMI [AOR = 7.36, 95 % CI (4.43-12.23)]. (Table 3)

## Discussion

Hypertension is becoming a major public health problem in Ethiopia. It contributes significantly to the high burden of cardiovascular diseases and premature mortality and morbidity [1]. This study tried to assess hypertension prevalence and its associated factors in ministry civil servants.

The overall prevalence of hypertension among ministry civil servants in this study was found to be 27.3 %. It was similar with that of studies done in Gondar city (28.3 %), Addis Ababa (30 %), Malaysia (27.8 %) and Nigeria (27.1 %) [8, 9, 15–17]. However, this finding is higher than that of studies done in southwest Ethiopia (13.2 %), Northwest Ethiopia (18.1 %) and Angola (23 %) [18–20]. This could be because the previous studies were done in both urban and rural dwellers where this study was conducted only in urban dwellers. The other reason might be due to the difference in study populations. In the southwest study, the study population was those who came for health care service in the hospitals. In addition, the other two studies were conducted in the populations.

However, the result of this study was lower than that of findings from Senegal (46 %) and Angola public sector workers (45.2 %) [19, 21]. The reason for this low prevalence of hypertension in our study as compared to the previous two studies might be the difference in socio-cultural and socio-economic status between the study populations.

Increased age was identified as a factor for hypertension in this study and many other studies [8, 9, 20, 22, 23]. Study participants older than 48 years were more likely to be hypertensive than those younger than 27 years. This could be due to the physiological change of blood vessels as the age is increased; in which blood vessels flexibility might be lost (hardening of the arteries) as age is increased.

History of smoking is one of the well-established risk factor for hypertension [24, 25]. Similarly in this study smokers were 2.34 times more likely to be hypertensive as compared to nonsmokers. The possible reason might be that cigarette smoking increases arterial inflammation and stiffness.

In this study, civil servants who were overweight/obese were 7.36 times more likely to be hypertensive as compared to civil servants with normal BMI. This might be due to the excess weight increasing blood cholesterol and triglyceride levels, and lowers high-density lipoprotein levels. This finding is in line with cross sectional study conducted in India, Nigeria, Senegal and North West Ethiopia [9, 20, 21, 24, 26].

Those who had family history of hypertension were 3.26 times more likely to be hypertensive. This result is in agreement with findings of some other studies in Northwest Ethiopia and Southwest Ethiopia [9, 27]. The possible reasons might be participants with family history of hypertension might have the same genetic components, and the fact that family tend to share the same lifestyle choices and behavior.

Civil servants who self-reported DM were 13.56 times more likely to be hypertensive. This finding is in line with some studies conducted in Senegal and Northwest Ethiopia [9, 21]. The high risk of hypertension among diabetic individuals could be due to increased peripheral vascular resistance and the fact that both diseases share common risk factors.

Unlike many other study reports [15, 19, 24, 26, 28], this finding did not revealed any association of hypertension with sex, education level, marital status, alcohol consumption, chat chewing, dietary habits, or physical activity. This could be explained by difference in study population, setting, sample size, socio economic and cultural difference between the two studies.

Possible limitations of this study include the cross sectional nature of the study which is impossible to establish temporal relationship between the hypertension and identified risk factors. The second limitation is that the study was conducted in an institution and this limits the generalizability of the finding to the whole population. Furthermore, the study was limited to behavioral and physical measurements and did not include biochemical. The method of data collection i.e., could bias the true value of the study.

## Conclusion

The prevalence of hypertension among federal ministry civil servants was found to be high; therefore, there is an indication for institution based hypertension-screening programs. Workplace wellness programs that consider the identified risk factors might help the prevention of hypertension in this population.
